# Kinematics characteristics of key point of interest during tennis serve among tennis players: a systematic review and meta-analysis

**DOI:** 10.3389/fspor.2024.1432030

**Published:** 2024-07-08

**Authors:** Julien Jacquier-Bret, Philippe Gorce

**Affiliations:** ^1^ International Institute of Biomechanics and Occupational Ergonomics, Hyères, France; ^2^ Université de Toulon, Toulon, France

**Keywords:** tennis player, tennis serve, kinematics, biomechanics, trophy position, racket low point, ball impact, worldwide analysis

## Abstract

The objective of this systematic review and meta-analysis was to provide an overview of kinematic parameters associated with key points of interest in the tennis serve. The research was conducted according to the PRISMA guideline without date restriction. Google scholar, Science Direct, PubMed/Medline, Mendeley, and Science.gov databases were scanned to find relevant studies. Only English peer-review original article focused on joint body angles at trophy position, racket low point and ball impact were retained. The review, quality appraisal, and data extraction from selected studies were performed independently by two reviewers. A meta-analysis was carried out on the most studied joint parameters. Among the 2,844 records identified, 27 articles were included. The wide variety of methods used required data homogenization for comparison purposes. Trunk inclination (25.0 ± 7.1°) and front knee flexion (64.5 ± 9.7°) were the most studied parameters for trophy position. Shoulder lateral rotation (130.1 ± 26.5°) was systematically evaluated for racket low point. At ball impact, shoulder elevation (110.7 ± 16.9°) and elbow flexion (30.1 ± 15.9°) were the most considered joint angles. The systematic review revealed that many kinematic parameters were not quantified at the various key points of interest. Knowledge of the kinematics is essential for understanding the gesture, implementing training methods, and improving the performance.

## 1 Introduction

The serve is considered the most important stroke in tennis. It allows the player to start a point by controlling the speed and direction of the ball to take the advantage over the opponent. Many studies have divided the tennis serve into phases based on key points. Early studies focused on ball impact, quantifying the biomechanical parameters of the upper limb (1). Other authors have assessed the posture at the instant of maximal knee flexion (2) or maximal shoulder external rotation (3, 4). In 2011, Kovacs and Ellenbecker (5) described the service through 3 phases with 8 stages: preparation phase (start, release, loading, and cocking), acceleration phase (acceleration and contact) and follow through phase (deceleration and finish). The postures identified in previous work have been summarized in 4 key points: (1) ball release (BR) which corresponds to the moment when the ball leaves the player's non-serving hand (5); (2) trophy position (TP) defined as the moment when the racket reaches its first peak of vertical movement (6) and corresponding to a fully loaded lower body position, i.e., elbow lowest vertical position and maximum knee flexion (5); (3) racket low point (RLP), corresponding to the moment of maximum shoulder rotation with tip of the racket head pointing toward the ground (5) coinciding with the lowest vertical position of the racket when it is behind the back (6); (4) ball impact (BI), corresponding to contact between the racket's sieve and the ball (5). Most of these key points have been studied using kinematic data over the last 30 years. All these works have studied the tennis serve by including a large number of parameters such as age, level, serve type, serve velocity, foot or finishing technique, etc., while considering only a few kinematic data. Elliott and Wood (7) studied the effect of foot technique, i.e., foot-up vs. foot-back, on shoulder, elbow, wrist, and hip and knee flexion in the sagittal plane at BI (7). Reid et al. (8) addressed the effect of age (3 groups) during a flat serve from trunk inclination (TP), shoulder flexion and rotation (RLP and BI) and elbow flexion (BI). Tubez et al. (9) also studied the effect of age (3 groups) from trunk and shoulder rotation as well as elbow, wrist, and knee flexion but only for TP. Liang et al. (10) compared 3 levels of middle-school female players by considering lower limb joint angles at jump take-off and landing. In 2023, Touzard et al. (11) studied the effect of racket size during a flat serve only through knee and ankle flexion (TP). Reid and Giblin (12) compare a normal and an arabesque flat serve during the follow-through phase. The authors only considered trunk inclination at BI but studied peak values for the shoulder, hip and knee in during the preparation phase and in the final position of the serve. Mourtzios et al. (13) studied the effect of the serve type (flat, slice and topspin) on knee and ankle flexion for TP and BI. This effect of serve, flat, vs. kick, vs. slice, was also studied by Sheet et al. 2011 on back, shoulder, elbow, and wrist trajectories over the entire serve (14). Tanabe and Ito (15) compared the contribution of different angular velocities of the upper limb during a slow vs. fast serve. A few studies have investigated a more exhaustive list of parameters to qualify the serve. In 2003, Fleisig et al. (3) considered 6 joint angles (trunk, elbow, wrist and knee flexion, trunk inclination and shoulder elevation) for RLP and 5 joint angles (trunk, elbow, wrist and knee flexion, and trunk inclination) for BI. Fett et al. (24) reported 13 kinematic parameters for TP (trunk axial rotation), RLP (trunk, elbow and knee flexion, trunk and shoulder rotation and trunk tilt) and BI (trunk, elbow, wrist and knee flexion, trunk tilt and shoulder elevation).

Despite all this work, there is no consensus on the kinematic values at the key points of interest in the serve, and yet they are essential for understanding the gesture, optimization, and performance or training. To our knowledge, only the work of Brito et al. has assessed the “biophysics” of the tennis serve throughout a systematic scoping review (16). The authors proposed a mapping of relevant information (age, sex, level, measurement tools, playing surface, type of serve) to highlight what is already known and to identify gaps in the literature. However, no synthesis of the kinematic parameters that describe the tennis serve has been proposed. Currently, the numerous works available in the literature present a great heterogeneity of data, calculation methods, definition and location, making it difficult to compare data. One of the main challenges will be to propose a range of valid kinematic data for the different key points of the tennis serve. Trainers and coaches are interesting in using this data to optimize player performance, education and training. In this context, this review would provide a global view of kinematic data for tennis service worldwide.

The objective was to propose a detailed systematic review of kinematic data for all joint angles at each key point of interest, i.e., trophy position, racket low point, and ball impact. To achieve this, a precise study of all the work was necessary, in order to homogenize the data in the same reference frame, in accordance with the convention of the International Society of Biomechanics. A meta-analysis was carried out on the most studied parameters in order to propose a mean value with its standard deviation.

## 2 Materials and methods

This study was conducted according to PRISMA guidelines for reporting systematic reviews and meta-analyses (17). The search has been performed between September and December, 2023.

### 2.1 Search strategy and eligibility criteria

The search strategy included five databases: PubMed/Medline, Science Direct, Mendeley, Science.gov, and Google Scholar. The following set of keywords was used in each database: “Tennis” AND “Serve” AND “Kinematics”. The search was limited to English-language, full-text, peer-reviewed tennis serve studies. No date restrictions have been set. Studies were excluded if: (1) the study design is reviews, systematic reviews, conference proceedings, books or book chapters, commentaries, case studies, and case series; (2) no numerical kinematics data is described; (3) kinematics data is not related to a tennis serve key point of interest; (4) kinematic data do not refer to the anatomical angles of the tennis player (racket, ball, etc.); (5) tennis players are beginners or suffer from an injury. No restrictions regarding age, sex, level (above beginner), and type of measurement tool to quantify kinematic data were applied.

**Figure 1:**
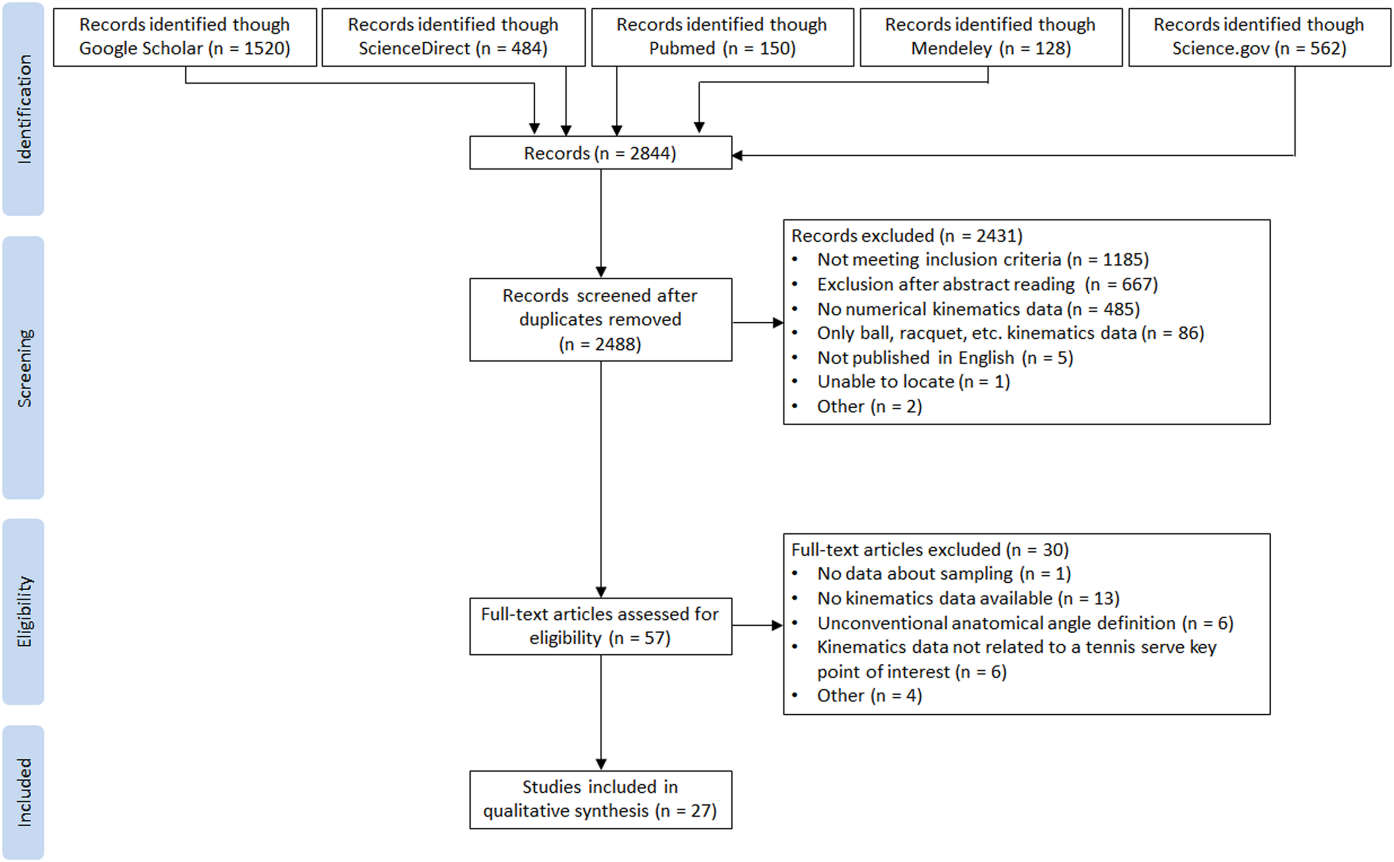
PRISMA flow chart.

Results were imported from all databases and compiled into a table. An automatic function was used to remove duplicates. All publications were separately screened by two reviewers (PG and JJB) for eligibility according to the inclusion and exclusion criteria. Each reviewer excluded studies that did not meet the criteria. All differences were resolved by consensus after a further reading of the articles. The search strategy, the selection process, and reasons for exclusion are presented in the PRISMA flow chart (Figure 1).

### 2.2 Methodological quality appraisal and risk of bias

Two reviewers independently assessed the quality of the 27 included studies using the modified CONSORT 2010 checklist (17). The evaluations were compared and discrepancies were discussed to provide the final decision after rereading the article. The classification of McFarland and Fischer (18) was used to provide the quality appraisal: (1) checklist items over 85% corresponded to high quality; (2) checklist items less than 50% meant low quality; (3) studies with checklist items between 50% and 85% were considered as medium quality.

### 2.3 Data extraction

The following data were extracted from the included articles: country, number of participants, participant characteristics (sex, age, height, weight, level of practice), type of tennis serve (flat, kick, slice, and topspin), foot position, equipment used for kinematic data acquisition, all available body kinematic angles per key point of interest during the tennis serve (trophy position, racket low point and ball impact).

## 3 Results

### 3.1 Search results

The search of the 5 databases collected 2,844 articles. After removing the 356 duplicates, 2,488 articles were checked. From title/abstract screening, 2,431 were excluded. Among the 57 remaining articles, 30 were excluded after full reading because they did not meet the inclusion criteria. Finally, 27 articles were included in the present literature review. The search process is shown in Figure 1.

### 3.2 Quality appraisal

Table 1 shows the quality appraisal of the 27 included studies. Twenty four articles were assessed with medium quality (50%–85% of items present in each study) and three were assessed as low quality.

**Table 1:** Quality appraisal of the included studies according to the modified CONSORT 2010 checklist.

High quality	Medium quality		Low quality
–	Abrams et al. (19)	Brocherie and Dinu (20)	Bingül et al. (21)
	Fadier et al. (22)	Fenter et al. (23)	Elliott et al. (1)
	Fett et al. (24)	Fleisig et al. (3)	Fadier et al. (25)
	Gillet et al. (26)	Hornestam et al. (27)	
	Konda et al. (28)	Mourtzios et al. (13)	
	Reid and Giblin (12)	Reid et al. (4)	
	Reid et al. (29)	Reid et al. (8)	
	Rogowski et al. (30)	Shafizadeh et al. (31)	
	Touzard et al. (11)	Tubez et al. (9)	
	Tubez et al. (32)	Wagner et al. (33)	
	Wang et al. (34)	Whiteside et al. (6)	
	Whiteside et al. (35)	Zappala et al. (36)	

### 3.3 Study characteristics

The 27 articles selected cover 4 of the 5 continents (America, Asia, Europe, and Oceania) in 12 different countries. Seven studies were conducted solely on male tennis players (19, 21, 23, 24, 26, 30, 33), three studies included only female players (6, 8, 35), nine studies had a mixed population (1, 3, 11, 13, 20, 22, 25, 31, 36) and height did not provide this information (4, 9, 12, 27–29, 32, 34). Different age categories were studied. Data were reported using the following designations: children, teenagers, prepubescent, pubescent, adults (ranging from 9.3 (25) to 34.4 (31) years). Several levels of expertise were also studied. Information was reported using the terms: professional, national, international, International Tennis number ranking (ITN), International Tennis Federation ranking (ITF), Women's Tennis Association ranking (WTA), and Collegiates.

The characteristics of the tennis serve were studied through the type (flat, kick, slice, and topspin), foot position (foot up, foot back), and side (ad or deuce). Reid and Giblin also studied the effect of the final position of the lower limb during the follow-through phase [normal vs. arabesque (12)]. Four other studies investigated the effect of a specific condition: difference between two laboratories (32), racket size (11), wearing a postural shirt vs. a normal shirt (36), and with or without opponent (31).

All kinematic data reported in the studies follow the 3 key points of interest proposed by Whiteside et al. (6, 35): Trophy Phase (TP) position, Racket Low Point (RLP) and Ball Impact (BI). The kinematic parameters considered for the neck and trunk are flexion/extension, axial rotation and relative inclination. Absolute inclination (Tilt) relative to the service line for the trunk has been added, as it has been taken into account in some works. For the shoulder, flexion, abduction, axial rotation and elevation have been reported. Only flexion was studied for the elbow, wrist, knee and ankle. Finally, pronation/supination of the forearm and axial rotation of the pelvis were reported in one and two studies respectively. Table 2 summarizes the characteristics of the 27 studies and the kinematic data by key point.

**Table 2:** Study characteristics and available kinematic data by key point of interest during tennis service for the 27 included studies.

Authors	Country	Study details				Tennis serve characteristics	Serve key point of interest	Neck			Trunk				Shoulder				Elbow	Forearm	Wrist	Pelvis	Hip	Knee	Ankle
								F	Incl	RA	F	Incl	RA	Tilt	F	Ab	RA	El	F	P/S	F	RA	F	F	F
Abrams et al. (19)	USA	N-participant	7	Age (year)	N/A	Flat, kick, slice serve	TP																		
		Male/Female	7M	Height (m)	N/A		RLP				X						X								
		Level of practice	Division I	Weight (kg)	N/A		BI																		
Bingul et al. (21)	Turkey	N-participant	15	Age (year)	18.4 ± 3.3	Flat serve	TP																		
		Male/Female	15 M	Height (m)	1.82 ± 0.06		RLP																		
		Level of practice	National/International	Weight (kg)	72.2 ± 7.9		BI									X			X		X				
Brocherie and Dinu (20)	France	N-participant	2	Age (year)	M:18; F:17	Flat serve	TP											X						X	
		Male/Female	1M/1F	Height (m)	M:1.77; F:1.73		RLP																		
		Level of practice	ITF category level 3–4	Weight (kg)	M:65; F:60		BI											X	X					X	
Elliott et al. (1)	Australia	N-participant	8	Age (year)	20.4	Male vs. Female	TP																	X	
		Male/Female	4M/4F	Height (m)	N/A		RLP																		
		Level of practice	National	Weight (kg)	N/A		BI								X				X		X		X	X	X
Fadier et al. (25)	France	N-participant	6	Age (year)	9.3 ± 0.8	Flat serve Deuce-side	TP																	X	X
		Male/Female	4M/2F	Height (m)	1.36 ± 0.06		RLP																		
		Level of practice	National	Weight (kg)	27.8 ± 3.8		BI																		
Fadier et al. (22)	France	N-participant	10	Age (year)	10.2 ± 1.4	Flat serve Deuce-side	TP																	X	X
		Male/Female	5M/5F	Height (m)	1.41 ± 0.09		RLP																		
		Level of practice	ITN 6–9	Weight (kg)	31.8 ± 6.7		BI																		
Fenter et al. (23)	USA	N-participant	9	Age (year)	19.6 ± 1.7	Multiple services during a 3-set match	TP																	X	
		Male/Female	9M	Height (m)	1.78 ± 0.04		RLP																		
		Level of practice	Division III	Weight (kg)	77.7 ± 7.5		BI																		
Fett et al. (24)	Germany	N-participant	14	Age (year)	14.6 ± 1.8	Flat serve Foot-up, foot-back	TP						X												
		Male/Female	14M	Height (m)	1.76 ± 0.16		RLP				X		X	X			X		X					X	
		Level of practice	National	Weight (kg)	61.4 ± 16.3		BI				X			X				X	X		X			X	
Fleisig et al. (3)	USA	N-participant	20	Age (year)	N/A	Deuce and ad-side	TP																		
		Male/Female	8M/12F	Height (m)	M: 1.83 ± 0.08; F: 1.74 ± 0.09		RLP				X						X	X	X		X			X	
		Level of practice	International	Weight (kg)	M: 77.6 ± 10.0; F: 62.2 ± 7.7		BI				X							X	X		X			X	
Gillet et al. (26)	France	N-participant	15	Age (year)	23.8 ± 3.4	Tennis serve with and without lower trapezius fatigue	TP								X	X	X								
		Male/Female	15M	Height (m)	1.83 ± 0.07		RLP								X	X	X								
		Level of practice	ITN 2–4	Weight (kg)	76.6 ± 8.7		BI								X	X	X								
Hornestam et al. (27)	Brazil	N-participant	32	Age (year)	SKF: 13.8 ± 1.0; GKF: 14.2 ± 1.2	Flat serve Smaller vs. greater knee flexion group	TP																	X	
		Male/Female	N/A	Height (m)	SKF: 1.66 ± 0.01; GKF: 1.67 ± 0.01		RLP																		
		Level of practice	ITN 5–7	Weight (kg)	SKF: 54.75 ± 6.25; GKF: 56.01 ± 6.69		BI																		
Konda et al. (28)	Japan	N-participant	20	Age (year)	21 ± 3	Flat serve	TP										X	X							
		Male/Female	N/A	Height (m)	1.74 ± 0.05		RLP										X								
		Level of practice	2 Pro, 18 collegiates	Weight (kg)	63.6 ± 6.1		BI																		
Mourtzios et al. (13)	Greece	N-participant	12	Age (year)	13.8 ± 1.22	Flat, slice, topspin serve Foot back	TP																	X	X
		Male/Female	6M/6F	Height (m)	1.66 ± 0.1		RLP																		
		Level of practice	N/A	Weight (kg)	55.2 ± 11.15		BI																	X	X
Reid and Giblin (12)	Australia	N-participant	8	Age (year)	17.3 ± 1.2	Flat serve Deuce-side Normal vs. arabesque serve	TP																	X	
		Male/Female	N/A	Height (m)	N/A		RLP																		
		Level of practice	International	Weight (kg)	N/A		BI							X											
Reid et al. (4)	Australia	N-participant	12	Age (year)	N/A	Flat, kick serve	TP																		
		Male/Female	N/A	Height (m)	1.83 ± 0.07		RLP					X					X	X						X	
		Level of practice	High-performance	Weight (kg)	79.9 ± 5.6		BI							X	X			X							
Reid et al. (29)	Australia	N-participant	12	Age (year)	N/A	Flat serve Deuce-side Foot-up, foot-back	TP					X													
		Male/Female	N/A	Height (m)	N/A		RLP																		
		Level of practice	High-performance	Weight (kg)	N/A		BI											X							
Reid et al. (8)	Australia	N-participant	28 (10PP, 10Pub, 8A)	Age (year)	PP: 10.5 ± 0.5; Pub: 14.6 ± 0.6; A: 21.5 ± 3.7	Flat serve	TP																		
		Male/Female	28F	Height (m)	PP: 1.44 ± 0.06; Pub: 1.67 ± 0.05; A: 1.69 ± 0.05		RLP					X					X								
		Level of practice	National and WTA	Weight (kg)	PP: 35.6 ± 2.9; Pub: 57.4 ± 3.9; A: 61.9 ± 4.3		BI					X			X	X			X						
Rogowski et al. (30)	France	N-participant	13	Age (year)	25.8 ± 5.0	Flat serve Deuce-side	TP								X	X	X								
		Male/Female	13M	Height (m)	1.8 ± 0.07		RLP								X	X	X								
		Level of practice	ITN 3	Weight (kg)	73.8 ± 9.3		BI								X	X	X								
Shafizadeh et al. (31)	United Kingdom	N-participant	10	Age (year)	34.4 ± 7.4	Mixed flat, slice and topspin serve With and without opponent	TP																		
		Male/Female	9M/1F	Height (m)	1.8 ± 0.08		RLP																		
		Level of practice	National	Weight (kg)	81.2 ± 13.3		BI	X	X	X	X	X	X			X									
Touzard et al. (11)	France	N-participant	9	Age (year)	9.9 ± 1.0	Flat serve R23, R25, R27 racquet size	TP																	X	X
		Male/Female	5M/4F	Height (m)	1.39 ± 0.07		RLP																		
		Level of practice	International	Weight (kg)	30.3 ± 5.1		BI																		
Tubez et al. (32)	Belgium	N-participant	28 (8C, 8 T, 8A)	Age (year)	C: 11.7 ± 0.9; T: 15.1 ± 1.4; A: 21.8 ± 3.1	Flat serve Deuce-side	TP						X				X	X	X		X			X	
		Male/Female	N/A	Height (m)	C: 1.48 ± 0.09; T: 1.75 ± 0.05; A: 1.81 ± 0.02		RLP																		
		Level of practice	ITN 1 to 6	Weight (kg)	C: 39.8 ± 8.5; T: 63.0 ± 7.0; A: 72.7 ± 5.3		BI																		
Tubez et al. (32)	Belgium	N-participant	13	Age (year)	22 ± 3	Flat serve Deuce-side	TP					X	X											X	
		Male/Female	N/A	Height (m)	1.80 ± 0.05		RLP																		
		Level of practice	ITN 3	Weight (kg)	71 ± 7		BI										X	X							
Wagner et al. (33)	Austria	N-participant	10	Age (year)	20.0 ± 4.0	Flat serve	TP																		
		Male/Female	10M	Height (m)	1.88 ± 0.07		RLP				X		X				X		X			X			
		Level of practice	National/International	Weight (kg)	77 ± 10		BI																		
Wang et al. (34)	Taiwan	N-participant	12	Age (year)	25.1 ± 3.7	Topspin serve with fatigue	TP				X	X	X			X	X		X	X	X				
		Male/Female	N/A	Height (m)	1.74 ± 0.07		RLP																		
		Level of practice	ITN 3	Weight (kg)	68.2 ± 9.7		BI				X	X	X			X	X		X	X	X				
Whiteside et al. (35)	Australia	N-participant	11 (5C, 5 T, 1Pro)	Age (year)	C: 10.6 ± 0.6; T: 14.8 ± 0.5; Pro: 26.7	Flat serve	TP					X		X											
		Male/Female	11F	Height (m)	C: 1.45 ± 0.06; T: 1.67 ± 0.04; Pro: 1.72		RLP										X								
		Level of practice	National and WTA	Weight (kg)	C: 35.8 ± 2.9; T: 50.8 ± 3.6; Pro: 65		BI					X		X				X	X						
Whiteside et al. (6)	Australia	N-participant	31 (12PP, 11Pub, 8A)	Age (year)	PP: 10.5 ± 0.5; Pub: 14.6 ± 0.7; A: 21.3 ± 3.8	Flat serve	TP					X		X											
		Male/Female	31F	Height (m)	PP: 1.43 ± 0.06; Pub: 1.70 ± 0.05; A: 1.69 ± 0.05		RLP										X								
		Level of practice	National and WTA	Weight (kg)	PP: 36.5 ± 3.7; Pub: 56.7 ± 3.8; A: 61.9 ± 4.2		BI					X		X				X	X			X		X	X
Zappala et al. (36)	USA	N-participant	9	Age (year)	M: 19.5 ± 1.3; F: 20.0 ± 0.71	N/A	TP																		
		Male/Female	4M/5F	Height (m)	M: 1.78 ± 0.05; F: 1.66 ± 0.04		RLP										X								
		Level of practice	Division III	Weight (kg)	N/A		BI																		

M, male; F, female; C, children; T, teenagers; A, adults; Pro, professionals; ITF, International Tennis Federation; ITN, International Tennis Number; WTA, Women's Tennis Association; PP, prepubescents; Pub, pubescents; TP, trophy position; RLP, racket low point; BI, ball impact; F, flexion; Incl, inclination; RA, axial rotation; Tilt, absolute inclination; El, elevation; SKF, smaller knee flexion; GKF, greater knee flexion.

Numerical kinematic values from the 27 included studies are presented by tennis serve key point of interest in Tables 3–5. Particular attention was paid to the data proposed in the studies, ensuring that they were defined in the same framework, following the International Society of Biomechanics' (ISB) conventions (37, 38). If this was not the case, a modification was applied to the data to make them homogeneous and therefore comparable with those of other studies. Two main cases were encountered: (1) when the authors reported intersegmental angular values, the complementary or supplementary angle was recomputed to correspond to the ISB definition, i.e., in the anatomical reference position (aligned segments) the joint angles are zero; (2) when the axes of rotation did not correspond to those of the ISB (notably for the shoulder and trunk), the movement was redecomposed to make the proposed values correspond to the ISB conventions.

**Table 3:** Mean (standard deviation) kinematic data reported for the trophy position.

Authors	Condition	Neck			Trunk				Shoulder				Elbow	Forearm	Wrist	Pelvis	Hip	Knee		Ankle	
		F	Incl	RA	F	Incl	RA	Tilt	F	Ab	RA	El	F	P/S	F	RA	F	Front F	Back F	Front F	Back F
Brocherie and Dinu (20)	Male											79.3 (5.5)						79.1 (6.1)	70.6 (9.1)		
	Female											78.1 (7.1)						78.5 (0.3)	73.2 (0.7)		
Elliott et al. (1)	Male																	52.2 (20.1)			
	Female																	53.9 (22.9)			
Fadier et al. (25)	–																	59.0 (12.0)	46.0 (10.0)	8.0 (9.0)	7.0 (5.0)
Fadier et al. (22)	–																	61.0 (10.0)	53.0 (21.0)	12.0 (6.0)	10.0 (6.0)
Fenter et al. (23)																		74.4 (13.0)			
Fett et al. (24)	Ad-side						120.5 (10.3)											69.5 (15.0)	75.0 (10.6)		
	Deuce-side						105.6 (9.5)											71.6 (15.7)	77.6 (9.6)		
Gillet et al. (26)	Without fatigue								0.5 (-)	78.0 (-)	60.0 (-)										
	With fatigue								3.0 (-)	78.0 (-)	60.0 (-)										
Hornestam et al. (27)	SKF																	55.6 (8.7)			
	GKF																	74.7 (5.9)			
Konda et al. (28)	–										63.0 (16.0)	74.0 (18.0)									
Mourtzios et al. (13)	Flat serve																	59.2 (17.8)	65.7 (14.5)	−6.8 (22.0)	−0.3 (22.3)
	Slice serve																	62.9 (19.4)	73.1 (15.7)	−8.7 (15.5)	−1.6 (25.9)
	Topspin serve																	66.2 (17.9)	82.8 (12.8)	−8.3 (19.0)	−19.8 (3.4)
Reid et al. (29)	Foot up				30.5 (6.4)																
	Foot back				31.1 (7.0)																
	Mini leg drive				32.1 (4.1)																
Reid and Giblin (12)	Normal serve					34.3 (7.6)												79.5 (8.2)	100.9 (21.0)		
	Arabesque serve					28.3 (10.9)												81.1 (8.1)	96.2 (4.8)		
Rogowski et al. (30)	–								18 (12)	66 (27)	76 (15)										
Touzard et al. (11)	R23																	55.0 (11.0)	52.0 (15.0)	9.0 (7.0)	9.0 (6.0)
	R25																	59.0 (9.0)	54.0 (16.0)	11.0 (8.0)	11.0 (8.0)
	R27																	60.0 (9.0)	53.0 (16.0)	9.0 (8.0)	11.0 (7.0)
Tubez et al. (32)	Prepubescent						4.0 (10.0)				62.0 (8.0)	75.0 (14.0)	107.0 (30.0)		10.0 (9.0)			47.0 (21.0)	45.0 (19.0)		
	Pubescent						9.0 (14.0)				57.0 (15.0)	67.0 (24.0)	85.0 (14.0)		16.0 (11.0)			68.0 (6.0)	64.0 (9.0)		
	Adult						15. (10.0)				27.0 (15.0)	88.0 (16.0)	88.0 (21.0)		2.0 (10.0)			63.0 (8.0)	62.0 (5.0)		
Tubez et al. (32)	Laboratory 1					14.5 (9.8)	20.6 (11.3)											65.0 (14.0)	79.0 (11.0)		
	Laboratory 2					17.6 (9.6)	19.0 (9.1)											64.0 (14.0)	76.0 (9.0)		
Wang et al. (34)	Expert				−15.4 (21.9)	26.5 (13.0)	15.5 (15.6)			56.8 (19.4)	111.1 (79.0)		98.9 (15.7)	5.5 (11.2)	6.4 (4.8)						
	Non-expert				16.8 (76.2)	10.4 (5.4)	27.3 (25.5)			60.7 (14.6)	91.0 (76.9)		77.8 (35.1)	12.9 (41.5)	12.0 (55.4)						
Whiteside et al. (35)	Children					29.0 (8.0)		34.0 (7.0)													
	Teenagers					23.0 (6.0)		46.0 (8.0)													
	Profesionnals					25.0 (3.0)		44.0 (1.0)													
Whiteside et al. (6)	Prepubescent					30.0 (7.0)		37.0 (12.0)													
	Pubescent					25.0 (6.0)		42.0 (7.0)													
	Adult					17.0 (11.0)		43.0 (7.0)													

F, flexion; Incl, inclination; RA, axial rotation; Ab, abduction; Tilt, absolute inclination; El, elevation; P/S, pronation/supination; R23-R25-R27, tennis racket size: scaled 23-inches, scaled 25-inches and full-size 27-inches; SKF, smaller knee flexion; GKF, greater knee flexion.

Negative values for “Trunk F” and “Ankles F” represent extension.

**Table 4:** Mean (standard deviation) kinematic data reported for the racket Low point.

Authors	Condition	Neck			Trunk				Shoulder				Elbow	Forearm	Wrist	Pelvis	Hip	Knee		Ankle	
		F	Incl	RA	F	Incl	RA	Tilt	F	Ab	RA	El	F	P/S	F	RA	F	Front F	Back F	Front F	Back F
Abrams et al. (19)	Flat serve				8.3 (5.5)						89.8 (-)										
	Kick serve				16.3 (4.9)						90.1 (-)										
	Slice serve				14.7 (5.0)						91.5 (-)										
Fett et al. (24)	Ad-side				44.0 (10.6)		126.7 (21.1)	19.2 (6.5)			138.1 (11.4)		132.2 (10.4)								
	Deuce-side				44.2 (1.3)		130.5 (19.8)	19.4 (5.8)			136.7 (10.6)		132.7 (9.8)								
Fleisig et al. (3)	-				66.0 (9.0)						172.0 (12.0)	101.0 (13.0)	104.0 (12.0)		66.0 (19.0)			13.0 (8.0)			
Gillet et al. (26)	Without fatigue								36.5 (-)	99.0 (-)	125.0 (-)										
	With fatigue								39.5 (-)	99.0 (-)	125.0 (-)										
Konda et al. (28)	-										137.6 (7.8)										
Reid et al. (4)	Flat serve					31.5 (7.3)					115.9 (18.3)	158.9 (8.5)									
	Kick serve					31.6 (7.5)					119.0 (18.3)	161.5 (10.2)									
Reid et al. (8)	Prepubescent					30.0 (6.0)					133.0 (13.0)										
	Pubescent					26.0 (6.0)					137.0 (8.0)										
	Adult					17.0 (11.0)					141.0 (7.0)										
Rogowski et al. (30)	-								15.0 (9.0)	94 (13.0)	132 (13.0)										
Wagner et al. (33)	-				39.0 (5.0)		102.0 (18.0)				61.0 (19.0)		112.0 (8.0)			99.0 (17.0)					
Whiteside et al. (35)	Children										152.0 (32)										
	Teenagers										138.0 (12)										
	Profesionnals										139.0 (1.0)										
Whiteside et al. (6)	Prepubescent										129.0 (12.0)										
	Pubescent										136.0 (9.0)										
	Adult										141.0 (7.0)										
Zappala et al. (36)	Normal shirt										172.0 (2.9)										
	Postural shirt										170.9 (3.7)										

F, flexion; Incl, inclination; RA, axial rotation; Ab, abduction; Tilt, absolute inclination; El, elevation; P/S, pronation/supination.

**Table 5:** Mean (standard deviation) kinematic data reported for the ball impact.

Authors	Condition	Neck			Trunk				Shoulder				Elbow	Forearm	Wrist	Pelvis	Hip	Knee		Ankle	
		F	Incl	RA	F	Incl	RA	Tilt	F	Ab	RA	El	F	P/S	F	RA	F	Front F	Back F	Front F	Back F
Brocherie and Dinu (20)	Male											150.3 (4.9)	10.7 (6.6)					19.6 (8.0)	10.8 (7.7)		
	Female											161.1 (1.3)	34.7 (4.0)					33.5 (4.6)	−0.2 (0.7)		
Bingul et al. (21)	-									104.7 (8.8)			39.8 (7.4)		28.7 (18.1)						
Elliott et al. (1)	Male								143.0 (4.9)				23.0 (12.3)		26.7 (7.5)		32.7 (4.0)	6.2 (2.5)		−36.8 (9.4)	
	Female								132.5 (19.4)				25.7 (15.6)		19.5 (3.1)		29.0 (4.5)	11.2 (2.2)		−29.8 (15.8)	
Fett et al. (24)	Ad-side				8.0 (9.6)			27.6 (4.4)				114.5 (6.4)	18.0 (8.5)		20.5 (6.9)			26.2 (6.4)	5.6 (8.1)		
	Deuce-side				7.6 (9.5)			27.2 (4.1)				114.0 (6.4)	18.0 (7.8)		20.3 (6.2)			29.1 (10.3)	6.1 (8.2)		
Fleisig et al. (3)	Male				48.0 (7.0)							101.0 (11.0)	20.0 (4.0)		15.0 (8.0)			24.0 (14.0)			
	Female				48.0 (7.0)							101.0 (11.0)	20.0 (4.0)		15.0 (8.0)			24.0 (14.0)			
Gillet et al. (26)	Without fatigue								40.0 (-)	96.0 (-)	73.0 (-)										
	With fatigue								43.0 (-)	96.0 (-)	69.0 (-)										
Reid and Giblin (12)	Normal serve							25.1 (7.4)													
	Arabesque serve							28.8 (6.6)													
Reid et al. (4)	Flat serve							41.7 (7.8)	56.4 (15.1)			108.9 (14.1)									
	Kick serve							33.4 (10.2)	67.2 (9.4)			107.7 (19.7)									
Reid et al. (29)	Foot up											107.7 (14.6)									
	Foot back											108.6 (15.2)									
	Mini leg drive											110.9 (12.4)									
Reid et al. (8)	Prepubescent					27.0 (9.0)			167.0 (14.0)	94.0 (11.0)			44.0 (12.0)								
	Pubescent					38.0 (8.0)			171.0 (14.0)	101.0 (11.0)			25.0 (10.0)								
	Adult					40.0 (6.0)			165.0 (6.0)	104.0 (13.0)			27.0 (8.0)								
Rogowski et al. (30)	-								7.0 (9.0)	103.0 (10.0)	76.0 (15.0)										
Shafizadeh et al. (31)	Without opponent	5.0 (-)	20.0 (-)	20.0 (-)	29.0 (-)	8.0 (-)	7.0 (-)			72.0 (-)											
	With opponent	8.0 (-)	22.0 (-)	30.0 (-)	33.0 (-)	10.0 (-)	5.0 (-)			77.0 (-)											
Tubez et al. (32)	Laboratory 1										117.5 (19.4)	105.0 (13.0)									
	Laboratory 2										120.3 (19.3)	106.0 (8.0)									
Wang et al. (34)	Expert				8.4 (17.0)	17.2 (10.7)	4.5 (10.9)			49.0 (13.2)	18.0 (41.4)		5.4 (7.8)	−7.2 (12.3)	5.3 (2.9)						
	Non-expert				12.8 (76.2)	5.3 (7.1)	28.9 (27.6)			61.7 (9.1)	126.7 (63.9)		79.9 (4.9)	−15.5 (42.3)	7.8 (56.3)						
Whiteside et al. (35)	Children					24.0 (10.0)						92.0 (9.0)	44.0 (13.0)								
	Teenagers					38.0 (10.0)						106.0 (8.0)	33.0 (5.0)								
	Profesionnals					48.0 (4.0)						108.0 (2.0)	39.0 (2.0)								
Whiteside et al. (6)	Prepubescent					25.0 (7.0)						95.0 (13.0)	42.0 (11.0)			94.0 (10.0)		18.0 (13.2)	15.9 (15.5)	−38.7 (16.9)	−37.8 (13.4)
	Pubescent					39.0 (8.0)						102.0 (10.0)	26.0 (11.0)			79.0 (10.0)		19.3 (10.8)	19.3 (17.5)	−46.2 (22.6)	−48.5 (18.3)
	Adult					40.0 (6.0)						104.0 (13.0)	27.0 (8.0)			75.0 (6.0)		15.8 (8.7)	13.9 (14.5)	−40.7 (15.1)	−41.4 (12.9)

F, flexion; Incl, inclination; RA, axial rotation; Ab, abduction; Tilt, absolute inclination; El, elevation; P/S, pronation/supination.

Negative values for ankle represent extension.

### 3.4 Trophy position

Table 3 reports the kinematic data on TP extracted from 18 studies. Compared with Table 2, two columns have been added for knees and ankles, as many authors distinguish between the front and back lower limb. They considered the knee and ankle flexion of the front lower limb (closest to the service line), respectively called front knee and front ankle flexion, and those of the rear limb (respectively called back knee and back ankle flexion). The most studied joint areas were trunk inclination and knees flexion (front and back), with 15 and 22 sets of data respectively extracted from 6 to 11 studies. Neck joint angles, hip flexion, and pelvis axial rotation were not studied. Other joints were less studied, with only a few data sets available (between 2 and 9).

**Figure 2:**
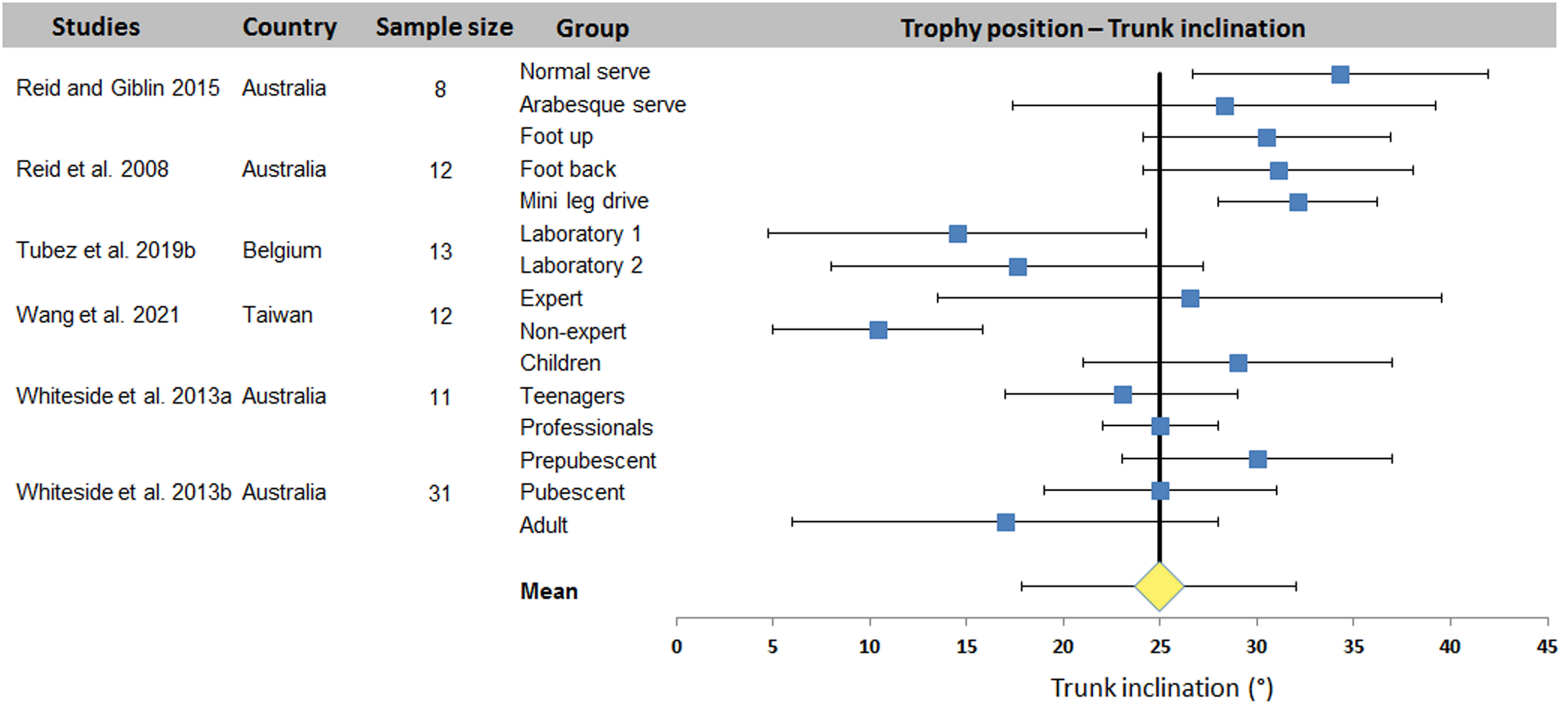
Distribution of trunk inclination values across studies. The square represents the mean value reported in each study. The diamond represents the mean trunk inclination computed over all studies. Horizontal bars represent standard deviation.

**Figure 3:**
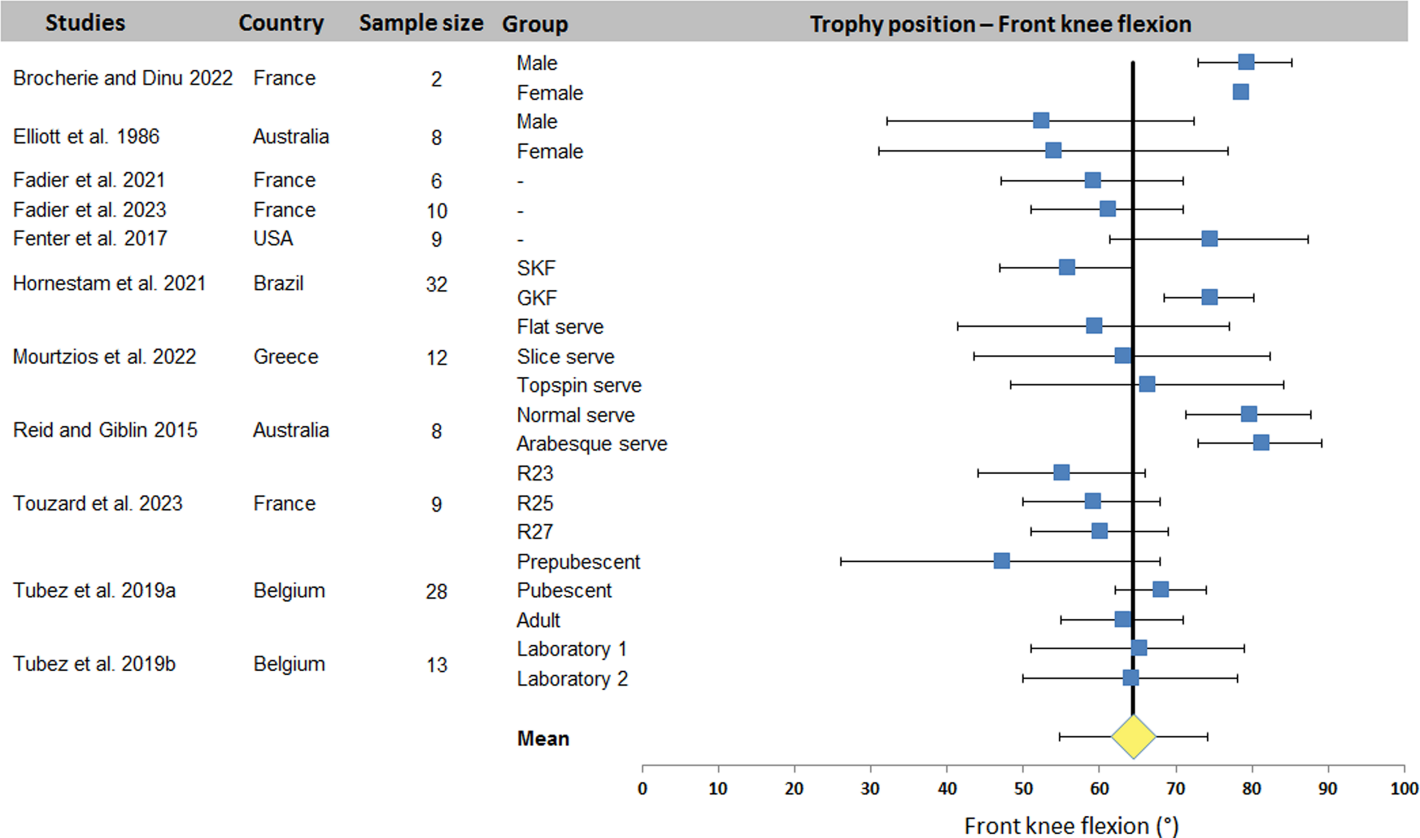
Distribution of front knee flexion values across studies. The square represents the mean value reported in each study. The diamond represents the mean front knee flexion computed over all studies. Horizontal bars represent standard deviation. R23-R25-R27, tennis racket size: scaled 23-inches, scaled 25-inches and full-size 27-inches; SKF, smaller knee flexion; GKF, greater knee flexion.

**Figure 4:**
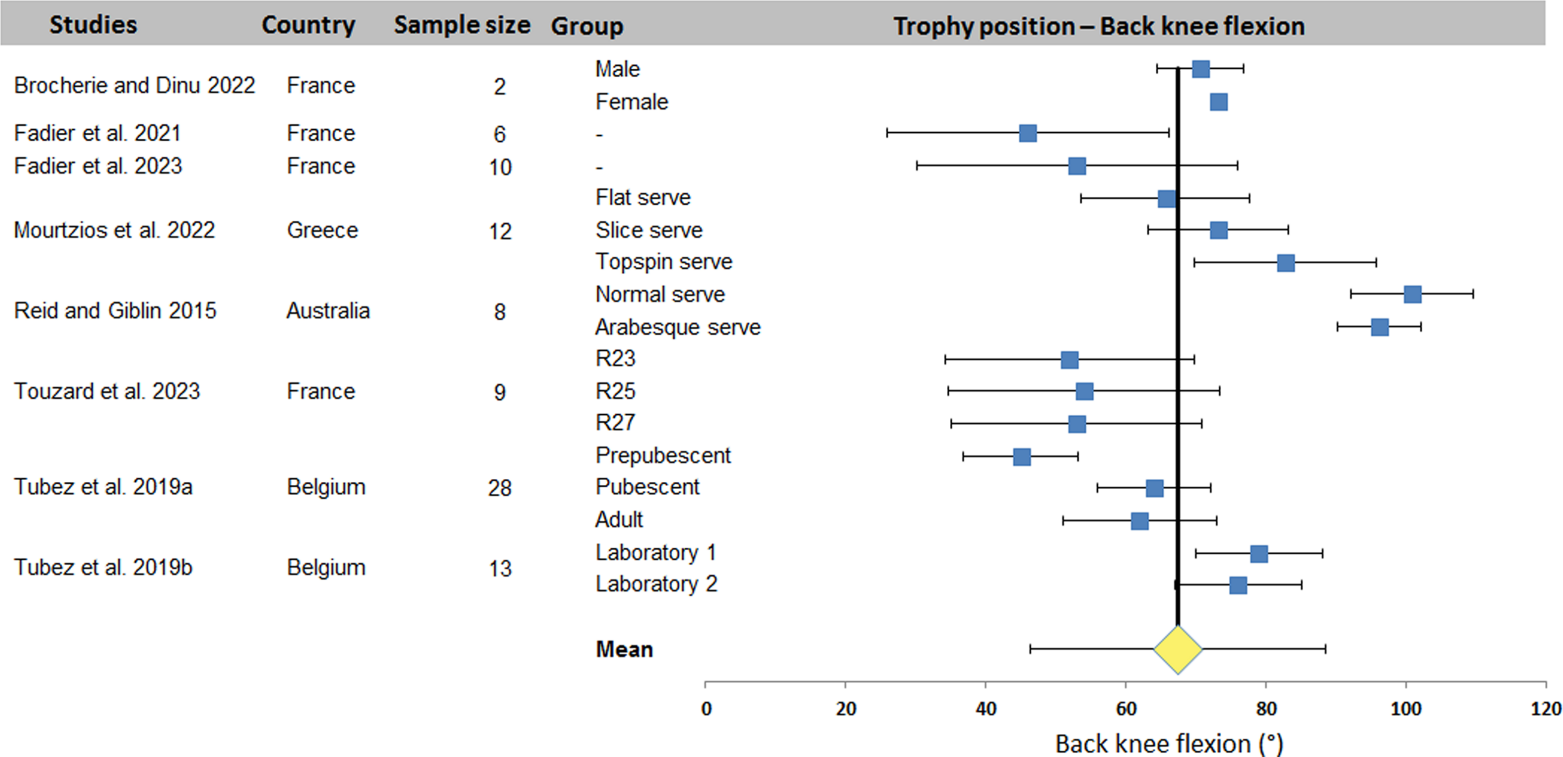
Distribution of back knee flexion values across studies. The square represents the mean value reported in each study. The diamond represents the mean back knee flexion computed over all studies. Horizontal bars represent standard deviation. R23-R25-R27, tennis racket size: scaled 23-inches, scaled 25-inches and full-size 27-inches.

Figures 2–4 display the distribution of values for the three most studied joint angles: trunk inclination, front knee and back knee flexion. A mean value (± standard deviation) was computed from the data of all studies: 25.0 ± 7.1° for trunk inclination, 64.5 ± 9.7° and 67.4 ± 16.4° for front and back knee respectively. The plots show a wide dispersion of data between subjects within a same study. On the other hand, some studies report less dispersion.

### 3.5 Racket low point

Table 4 summarizes values of kinematic data on RLP extracted from 12 studies. Shoulder axial rotation was systematically measured (24 sets of data from the 12 studies). Neck joint angle, forearm pronation/supination, and hip and ankle flexions were not studied. Other joints were addressed in 1 or 4 studies (1–7 data sets).

**Figure 5:**
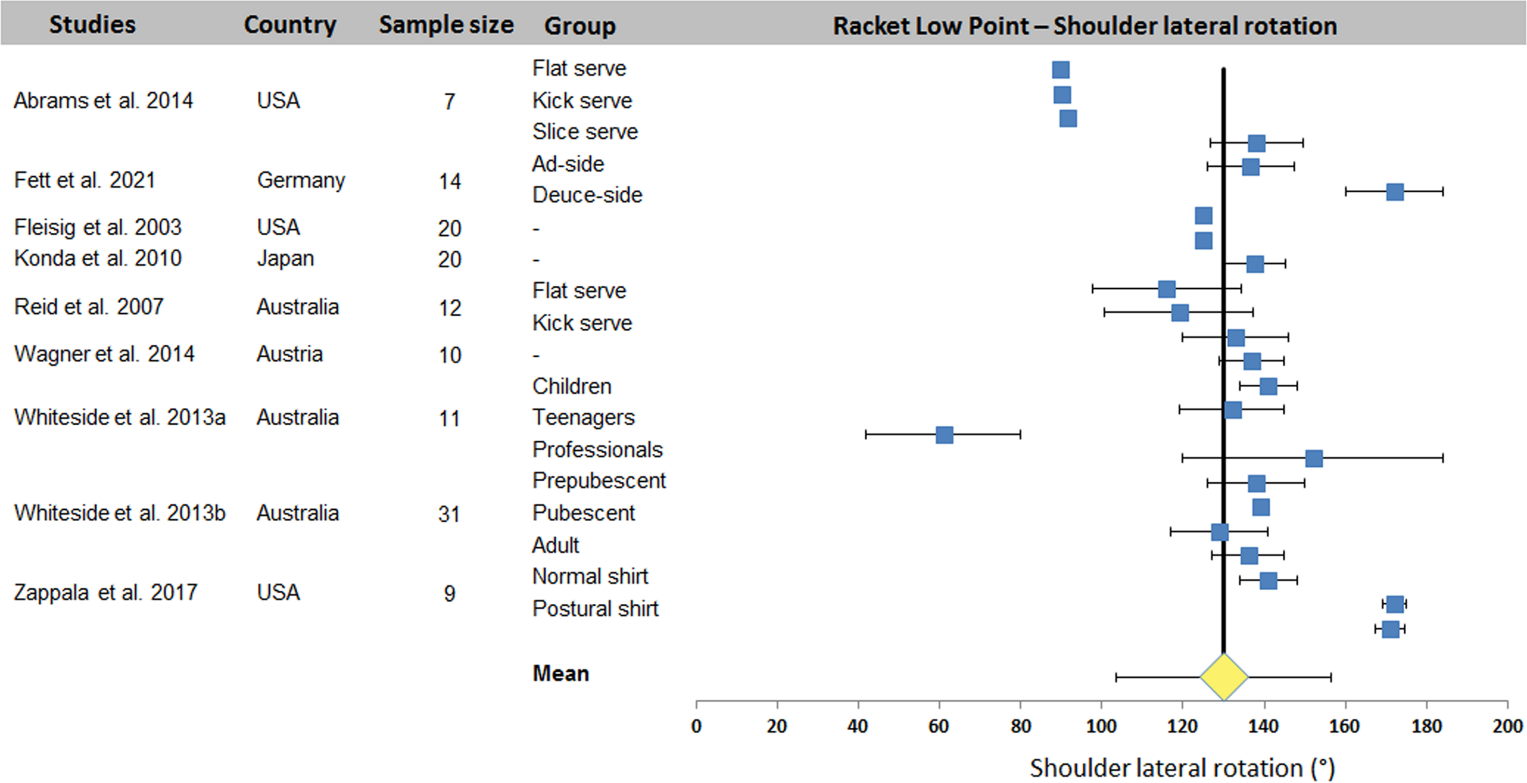
Distribution of shoulder lateral rotation values across studies. The square represents the mean value reported in each study. The diamond represents the mean shoulder external rotation computed over all studies. Horizontal bars represent standard deviation.

Figure 5 presents the distribution of values for the shoulder lateral rotation. A mean value of 130.1 ± 26.5° was found across studies. The dispersion is smaller than that observed for the angles studied during the TP. Only Wagner et al. study (33) and the Whiteside et al. (6) studies have significant dispersion. It should be noted that Abrams et al. (19) work did not propose standard deviation values.

### 3.6 Ball impact

Table 5 diplays kinematic data from the 16 ball impact studies. Shoulder elevation and elbow flexion were the most studied joint angles (8 and 9 studies with 19 and 20 data sets respectively). Other joint angles were reported by 1–6 studies (2–13 data sets).

**Figure 6:**
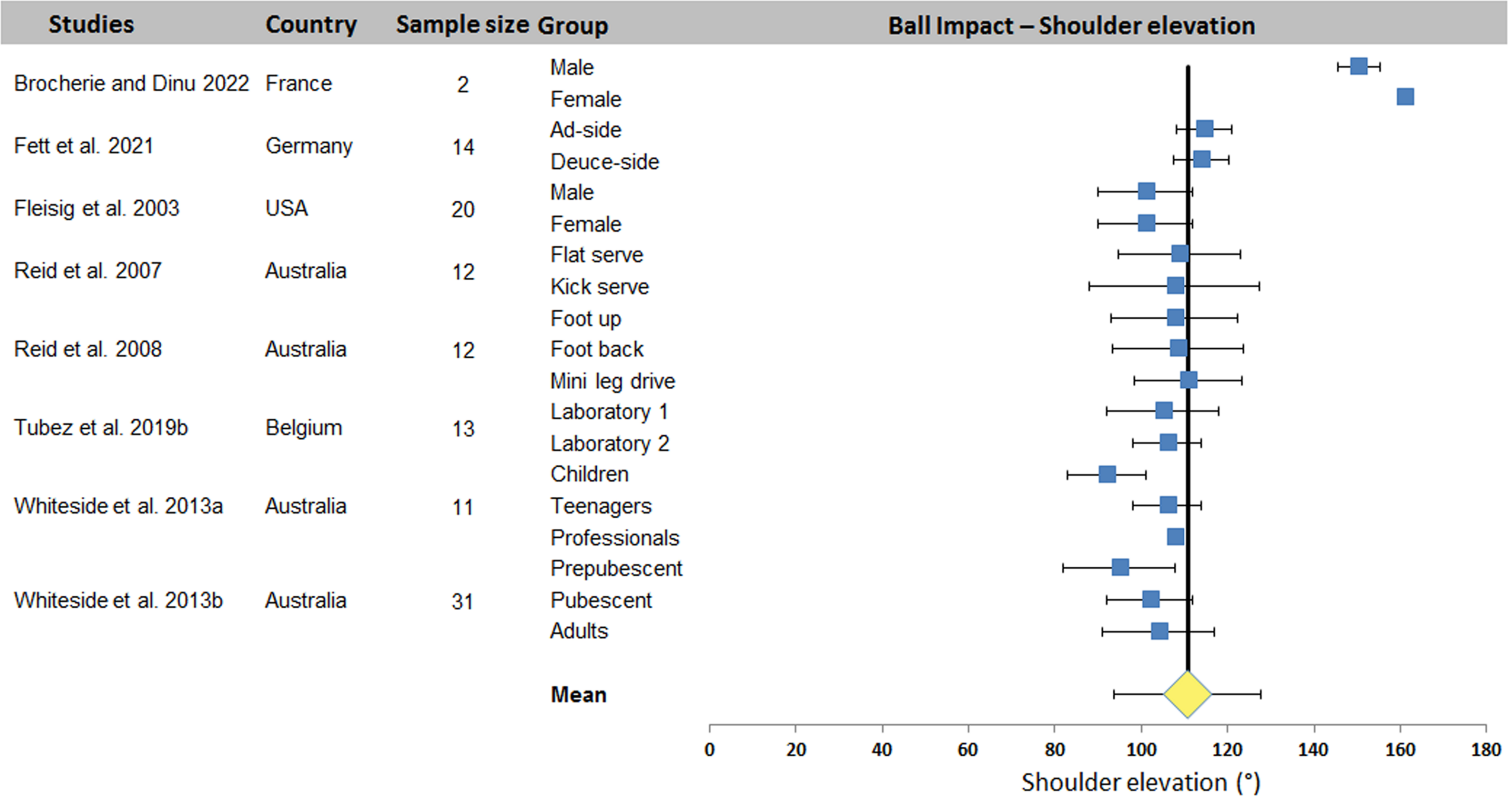
Distribution of shoulder elevation values across studies. The square represents the mean value reported in each study. The diamond represents the mean shoulder elevation computed over all studies. Horizontal bars represent standard deviation.

**Figure 7:**
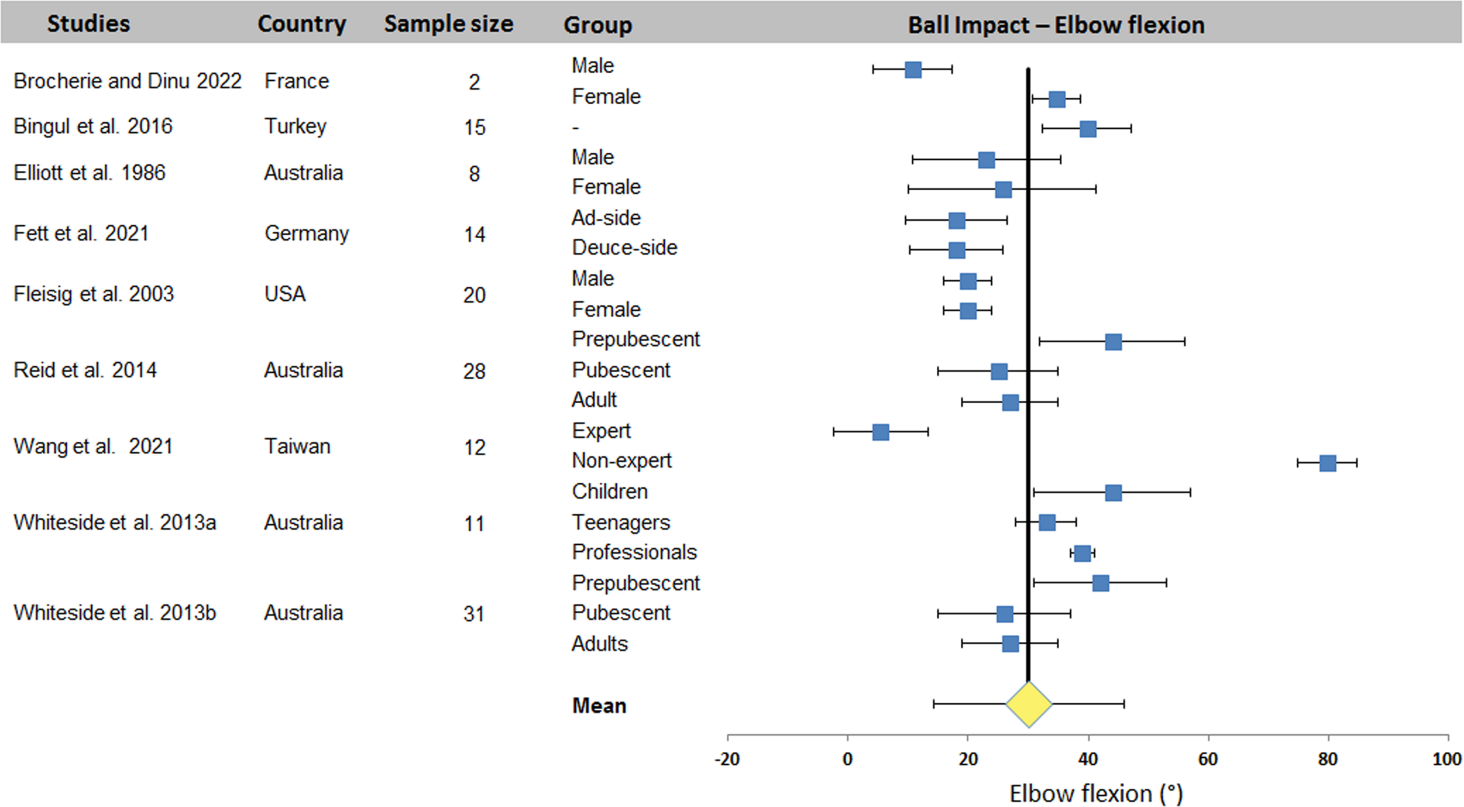
Distribution of elbow flexion across studies. The square represents the mean value reported in each study. The diamond represents the mean elbow flexion computed over all studies. Horizontal bars represent standard deviation.

Figure 6 shows the 22 series of shoulder elevation data. Except the data from Brocherie's study (20), the observed values show relatively low variability, with acceptable homogeneity between studies despite the very different experimental conditions. A mean value of 110.7 ± 16.9° was computed for all 9 studies. Elbow flexion, on the other hand, showed greater extra- and intra-study dispersion (Figure 7). A mean value of 30.1 ± 15.9° was computed across the 10 studies.

## 4 Discussion

The aim of this study was to carry out a systematic review and meta-analysis of kinematic data on the tennis serve. One of the underlying questions is which joint angles are studied in relation to which key points, regardless of player characteristics (age, sex, level), methods of gesture analysis (video cameras, inertial measurement units, optoelectronic cameras, etc.) and the conditions under which the serve is studied (type of serve, foot technique, side of serve, etc.).

One general result that emerges clearly from this review is that not all the kinematic parameters that help define the player's body movement have been determined during the serve, i.e., all the joint angles. Summary tables were used to identify the joints studied in the selected studies (Table 2) and to quantify them according to each key point in each anatomical plane (Tables 3–5). The meta-analysis proposed mean values according to key points for 7 joint angles: trunk inclination (6 studies), front knee flexion (11 studies) and back knee flexion (8 studies) for TP, shoulder lateral rotation (9 studies) for RLP, and shoulder elevation (8 studies) and elbow flexion (9 studies) for BI. The data set presented is the result of data homogenization (system of common homogenous references in compliance with ISB convention).

The key point approach is often used in the literature. This could be explained by the complexity of the movement and the need for highly sophisticated measuring equipment to quantify whole-body motion during the full serve. Very few studies have proposed the temporal evolution of joint angles for all phases of the tennis serve (5). Reid et al. (4) and more recently Fett et al. (24) equipped all player segments (62 and 86 reflexive markers) and recorded movement using optoelectronic cameras. However, only a few parameters have been studied and temporal evolutions have hardly ever been exploited. Shafizadeh et al. (31) proposed a temporal profile of neck, back and shoulder abduction angles. The majority of studies have therefore used postures at various key points to study the tennis serve and some differences have been identified.

### 4.1 Characterization of key points during tennis serve

The analysis showed that there was a widespread consensus regarding the definition of serve key points, i.e., ball release, trophy position, racket low point, and ball impact. Trophy position was defined by Whiteside et al. (6) as the “first peak vertical displacement of the racket”. Kovacs and Ellenbecker (5) defines this moment as the “fully loaded lower body position, i.e., elbow lowest vertical position and maximum knee flexion”. Reid et al. (4) specifies that this position is characterized only by the knee flexion. Racket low point has been defined as the lowest vertical position of the racket when it is behind the back (6). Kovacs completes this definition by adding that this instant corresponds to maximal shoulder external rotation coinciding with the tip of the racket head pointing toward the ground (5). Several authors have therefore equated this key point with the moment of maximum external shoulder rotation (4, 19). The authors all agree that Ball Impact is the instant of contact between the ball and the racket. These key points have been used to define the different phases of the serve. There are two approaches. One considers that a phase is defined between two key points. Three phases are then described: preparation, propulsion and forwardswing (6, 24). The second approach is also divided into 3 phases with 8 stages: preparation phase (4 phases: start, release, loading, and cocking), acceleration phase (2 phases: acceleration and contact) and follow through phase (2 phase: deceleration and finish) (5, 20). In this framework, the definition of the three key points presented in this review, i.e., TP, RLP and BI, has taken into account the great variability in the names and parameters associated with these key points to construct summary Tables 2–5.

### 4.2 Different methods for defining and measuring joint angles between studies

Completing Tables 2–5 required particular care in determining the kinematic values proposed. These are directly linked to the measurement methods and conventions used to define the various computational references. This is despite the existence of an international convention published by the International Society of Biomechanics (37, 38) to define all anatomical landmarks. Several difficulties were encountered. Firstly, several authors used different vocabulary to define the same joint angle. The most common example is trunk inclination.

Several terms have been used: “lateral flexion” (31), “trunk tilt” (6), and “lateral flexion shoulder-pelvis alignment separation angle” (4). A wide disparity was also observed in the definition of shoulder angles. Terms such as horizontal flexion (8), horizontal shoulder extension (9), upper arm-thorax elevation angle (4), shoulder horizontal adduction (3), or simply “shoulder angle” (21) make it difficult to interpret values. Only medio-lateral rotation has been correctly defined using the terms external and internal shoulder rotation. Another difficulty was encountered with the shoulder. In the landmark-based computation method, the choice of rotation sequence has a direct influence on the rotation angle values obtained (39). As a result, several angles cannot be compared due to this difference in computation technique.

On the other hand, some angle definitions are defined in relation to different references, which make it impossible to compare specific angles with others. For example, we find trunk tilt for the rotation of the trunk in relation to the pelvis, and upper torso position for the angle between the trunk and the baseline, measured in the absolute reference (24). There were also variations in angle measurements. According to the ISB convention, all angles are defined as zero in the anatomical reference position. However, some authors considered the direct value separating two consecutive segments, resulting in a value of 180° when the two segments are aligned. This problem has been encountered for elbows (21), knees (13, 20) and ankles (35), requiring data transformation in order to make comparisons with other articles.

Finally, when a rotation was expressed in a non-conventional frame of reference or by the absence of a direction of rotation, modifications were made to homogenize the data.

### 4.3 Data heterogeneity

The systematic review identified 27 articles in which a total of 18 kinematic parameters were quantified at different key point of interest in the tennis serve. Results (Table 2) showed that Wang et al. (34) reported the highest number of data for one phase (8 joint angles) and also the highest number of parameters in one study with 16 values (8 for TP and 8 for BI). Fett et al. (24) and Fleisig et al. (3) rank second and third respectively, with 13 and 11 kinematic parameters studied, including the maximum for RLP (6 joint angles). Table 2 also shows that trunk inclination, shoulder rotation and elevation, elbow flexion and knee flexion were the most studied joints, regardless of experimental conditions. These 5 joint angles were retained for a meta-analysis. The results were presented in the form of forest plots, showing the mean and standard deviation of each condition in each study, as well as the mean over all the included studies. For TP, a mean of 25.0 ± 7.1° was found for trunk inclination, 64.5 ± 9.7° for front knee and 67.4 ± 16.4° for back knee flexion. For RLP, a mean of 130.1 ± 26.5° was obtained for shoulder external rotation. Finally, a mean of 110.7 ± 16.9° and 30.1 ± 15.9° were computed for shoulder elevation and elbow flexion respectively for BI.

For TP, the results show a low overall variability for trunk and front knee flexion. Other variability ranges from 20° to 50°. These higher variabilities are the consequence of one or two studies that have a significant impact on dispersion. This is true of the studies by Brocherie and Dinu (20) for shoulder elevation and Wang et al. (34) for elbow flexion during BI. The relative dispersion of each study shows variability (between 2 and 60° dispersion). Under these conditions, the smaller the number of studies included in the meta-analysis, the greater the effect of the dispersion of each study on the overall variability of each kinematic parameter studied. In our case, the parameter with the most data, i.e., shoulder axial rotation, only provided 22 quantified data sets for 16 studies.

The wide variability observed between studies is due to the wide range of populations evaluated and the experimental conditions under which the tennis serve was carried out. Indeed, a large variability in age [children aged 9 (11) vs. pubescent (4), vs. adult (31)] and level [national (24) vs. international (3)] was observed between the included studies (for both sexes). On the other hand, the type of serve, the foot technique, the side of the serve, the conditions of execution (match or laboratory, with a target or not, with an opponent or not, state of fatigue) are all parameters that enhance the heterogeneity of the results. Despite this heterogeneity, the analysis has enabled us to propose an average value for trunk inclination and front knee flexion, with a dispersion of less than 20°, which can be used in training, education and optimization. To these two parameters, we could add shoulder elevation and elbow flexion, which would have an equivalent dispersion without the studies that present very different results without any particular justification. The mean values would change from 110.7 ± 16.9° and 30.1 ± 15.9° to 104.6 ± 6.1° and 29.2 ± 9.9° respectively, making them more relevant.

### 4.4 Application of key findings

The meta-analysis enabled to propose, for each key point of the serve, the joint angles encountered in the greatest number of studies to quantify a sufficiently relevant and homogeneous mean value. The results were: 25.0 ± 7.1° for trunk inclination, 64.5 ± 9.7° for front knee flexion, and 67.4 ± 16.4° for back knee flexion during TP; 130.1 ± 26.5° for shoulder lateral rotation during RLP; and 110.7 ± 16.9° for shoulder elevation and 30.1 ± 15.9° for elbow flexion during BI. Hornestam et al. (27) showed that knee flexion had an impact on serve performance. Indeed, the group with the lowest knee flexion, 10° less than that presented in the present study (55.6 ± 8.47°), had a racket velocity reduction of 3.33 km.h^−1^. A recent study also showed that knee flexion was correlated with racket velocity, indicating that greater flexion results in greater racket velocity (40). It would therefore be advisable to look for significant knee flexion during TP in order to optimize racket velocity during the serve. The search for significant lateral shoulder rotation also seems to be important at RLP (8, 24). In fact, this rotation is directly correlated to the racket velocity (40). In addition, a high lateral rotation generates a very high medial rotation velocity, which contributes 40%–50% to the racket linear velocity (15, 41). At BI, shoulder elevation and low elbow flexion contribute to ball impact at high altitudes above 2.5 m (6, 24). Many authors have reported elbow flexion close to 30° at BI (1, 35). A slightly flexed elbow seems to have a mechanical advantage for the medial rotation of the shoulder and therefore the racket velocity (42).

### 4.5 Limitations

Some limitations should be addressed. The first limitation concerns data acquisition. Very different measurement tools were used to quantify the kinematic parameters. This implies a different level of precision which could have an incidence on the values obtained. A second limitation concerns the small sample size (2–32) and the low number of repetitions (<5), which are not statistically representative of the population. Another limitation concerns the selection of studies through inclusion criteria (limited to “original article” written in English). This could have led to exclude or omit interesting works that could have completed and extended the results of the present review and meta-analysis.

## 5 Conclusion

The present systematic review and meta-analysis identified trunk inclination, shoulder elevation and lateral rotation, elbow flexion and knee flexion as the most studied kinematic parameters at the various key points of interest in tennis serve. A mean value with a standard deviation has been proposed for each of them. More work needs to be carried out in the future, taking into account as many joint angles as possible, in order to obtain more data on the complete kinematics of the various key tennis serve postures. The full set of kinematic parameters is essential for a precise understanding of the tennis serve motion, and for their use in training, coaching and performance optimization.
